# Supplementary material for: Biotransformation of Penindolone, an Influenza A Virus Inhibitor
Source: Molecules. 2023 Feb 3;28(3):1479. doi: 10.3390/molecules28031479 (PMC9920254; doi:10.3390/molecules28031479)
Supplement: Supplementary file 1 [file molecules-28-01479-s001.zip › molecules-2174988-supplementary.pdf]

Biotransformation of Penindolone, an Influenza A Virus Inhibitor

Shuai Liu [1,†], Keming Zheng [1,†], Yilin Jiang [1], Susu Gai [1], Bohan Li [1], Dehai Li [1,2], Shuang Yang [1,2,*] and Zhihua Lv [1,2,*]

[1] Key Laboratory of Marine Drugs, Ministry of Education of China, School of Medicine and Pharmacy, Ocean University of China, Qingdao 266003, China; liushuai4775@stu.ouc.edu.cn (S.L.); zhengkeming@stu.ouc.edu.cn (K.Z.); jiangyilin@stu.ouc.edu.cn (Y.J.); gss@stu.ouc.edu.cn (S.G.); libohan1228@stu.ouc.edu.cn (B.L.); dehaili@ouc.edu.cn (D.L.)

[2] Laboratory of Marine Drugs and Bioproducts, Pilot National Laboratory for Marine Science and Technology, Qingdao 266237, China

* Correspondence: yangshuang@ouc.edu.cn (S.Y.); lvzhihua@ouc.edu.cn (Z.L.)

† These authors contributed to the work equally.

Contents

Tables

Table S1. Reaction conditions for CYPs and UGTs probe substrates. 3
Table S2. Ion mode and MRM parameters for determining metabolites of CYPs probe substrates. 3
Table S3. Mobile phase composition for determining metabolites of CYPs probe substrates in ESI (+) mode. 4
Table S4. Mobile phase composition for determining metabolites of CYPs probe substrates in ESI (–) mode. 4
Table S5. Ion mode and mode and MRM parameters for metabolites of UGTs probe substrates. . 4
Table S6. Mobile phase composition for determining metabolites of UGTs probe substrates. 4

Figures

Figure S1. Chemical structures of penindolone and its derivatives HDYL-GQQ-2399, QL-Vir-09 and lbh3-78-2. 5
Figure S2. MS^2 spectra of M0 (**a-1**), M3 (**b-1**), M4 (**c-1**), M5 (**d-1**) obtained in negative mode and M0 (**a-2**), M3 (**b-2**), M4 (**c-2**), M5 (**d-2**) obtained in positive mode. 5
Figure S3. MS^2 spectra of M1 (**a-1** obtained in negative mode, **a-2** obtained in positive mode), M2 (**b-1** obtained in negative mode, **b-2** obtained in positive mode), M6 (**c**), M7 (**d**), M14 (**e**) and M15 (**f**). 6
Figure S4. MS^2 spectra of M8. 6
Figure S5. MS^2 spectra of M9 (**a**), M10 (**b**), M16 (**c**), M17 (**d**) and M18 (**e**). 7
Figure S6. MS^2 spectra of M11 (**a**) and M19 (**b**). 7
Figure S7. MS^2 spectra of M12 (**a**), M13 (**b**), M20 (**d**), M21 (**e**) and M22 (**f**). 8
Figure S8. MS^2 spectra of M23 (**a**), M24 (**b**) and M25 (**c**). 8
Figure S9. The validation of rat (**a**, **d**), mouse (**b**, **e**) and human (**c**, **f**) LMs system was confirmed by specific probe substrates. Error bars represent standard deviation. 9
Figure S10. Enzyme (CYPs and UGTs) kinetic plots of PND metabolism in rat (**a**, **d**), mouse (**b**, **e**) and human (**c**, **f**) LMs. Data are shown as Michaelis-Menten plots. The insert shows the Lineweaver-Burk plot of 1/[S (μM)] versus 1/[V (nmol/min/mg protein)]. 9

Supplementary Section: Quantification for Metabolites of Probe Substrates

The metabolites, incubation concentration and time of CYPs/UGTs probe substrates shown in Table S1. The separation of CYPs specific substrates metabolites were achieved on UltiMate 3000 UHPLC systems (Thermo Fisher Scientific, MA, USA) using an Agilent ZORBAX Eclipse plus C_{18} column (2.1 × 50 mm, 3.5 μm) and the column temperature was kept at 25 °C. The detection of analytes and IS_1 (propranolol or paeonol) was performed using a TSQ Quantiva™ triple quadruple mass spectrometer (Thermo Fisher Scientific, MA, USA) operating in the positive and negative ion mode with an ESI source. The ion transitions and MS/MS parameters for probe substrates metabolites and IS_1 (propranolol and paeonol) as follows: spray voltage (static), ion transfer tube temperature (325 °C), vaporizer temperature (275 °C), sheath gas (35 Arb), aux gas (10 Arb), CID gas (2 mTorr) and dwell time (20 ms). The precursor-to product ion pair and the collision energy (CE) were shown in Table S2. The sample injection volume was 5 μL and mobile phase composition and gradient elution shown in Table S3 and Table S4.

The UPLC–MS/MS determination of the UGTs specific substrates metabolites were carried out using a Xevo TQ-XS triple quadrupole mass spectrometer equipped with a UPLC H-Class PLUS system. The analytical column used was an ACQUITY UPLC BEH C_{18} column (2.1 × 50 mm, 1.7 μm; Waters, MA, USA) with a C_{18} Vanguard Pre-Colum (2.1 × 5 mm, 1.7 μm; Waters, MA, USA). Both column oven and autosampler were maintained at 25 °C. Mass spectrometry with an electrospray ionization (ESI) source was operated in negative and positive modes for detection. The capillary voltage and desolvation temperature were set at –40 or 58 KV and 500 °C, respectively. The desolvation gas and cone gas (nitrogen) were delivered at 1000 L/Hr and 150 L/Hr, respectively. The collision gas (argon) flow was set at 0.15 mL/min. The multiple reaction monitoring (MRM) mode was employed for the determination with parent-to-daughter ion transitions of substrates metabolite and IS_2 (phenacetin and chlorpropamide) as shown in Table S5. The sample injection volume was 2 μL and mobile phase composition and gradient elution were shown in Table S6.

Supplementary Tables

Table S1. Reaction conditions for CYPs and UGTs probe substrates.

Isoforms	Substrates (Conc.)	Metabolite	Incubation time (min)
CYP1A2	Phenacetin (50μM)	Acetaminophen	20
CYP2C11	Mephenytoin (50μM)	4-Hydroxymephenytoin	20
CYP2D2/6	Dextromethorphan (10μM)	Dextrophan	10
CYP2C6/9	Diclofenac (10μM)	4-Hydroxydiclofenac	10
CYP3A4	Midazolam (10μM)	1-Hydroxymidazolam	10
CYP2E1	Chlorzoxazone (100μM)	6-Hydroxychlorzoxazone	10
UGT1A1	Estradiol (2μM)	β-estradiol-3-glucuronide	60
UGT1A3	Chenodeoxycholic acid (50μM)	Chenodeoxycholic acid 24-acyl-β-D-glucuronide	60
UGT1A4	Trifluoperazine (50μM)	Trifluoperazine N-Glucuronide	60
UGT1A6	Serotonin (20μM)	Serotonin-β-D-glucuronide	60
UGT1A9	Propofol (2μM)	Propofol-β-D-glucuronide	60
UGT2B7	Zidovudine (200μM)	3′-azido-3′-deoxythymidine-β-D-glucuronide	60

Table S2. Ion mode and MRM parameters for determining metabolites of CYPs probe substrates.

Isoforms	Metabolite	Ion mode	Precursor (*m*/*z*)	Product (*m*/*z*)	Collision Energy （eV）
CYP1A2	Acetaminophen	ESI (+)	152.1	110.1	16
CYP2C11	4-Hydroxymephenytoin	ESI (+)	235.1	150.1	18
CYP2D2/6	Dextrophan	ESI (+)	258.2	157.1	37
CYP2C6/9	4-Hydroxydiclofenac	ESI (+)	312.3	230.0	30
CYP3A4	1-Hydroxymidazolam	ESI (+)	342.1	203.0	26
IS_1 (+)	Propranolol	ESI (+)	260.0	183.0	17
CYP2E1	6-Hydroxychlorzoxazone	ESI (–)	184.0	120.0	21
IS_1 (–)	Paeonol	ESI (–)	227.0	143.6	29

Table S3. Mobile phase composition for determining metabolites of CYPs probe substrates in ESI (+) mode.

Time(min)	Water-0.1% FA	MeOH-0.1% FA
0.00	95	5
1.50	95	5
1.51	5	95
4.50	5	95
4.51	95	5
6.00	95	5

Table S4. Mobile phase composition for determining metabolites of CYPs probe substrates in ESI (–) mode.

Time(min)	water	Acetonitrile
0.00	95	5
1.50	95	5
1.51	5	95
3.50	5	95
3.51	95	5
5.00	95	5

Table S5. Ion mode and mode and MRM parameters for metabolites of UGTs probe substrates.

Isoforms	Metabolite	Ion mode	Parent (*m*/*z*)	Daughter (*m*/*z*)	Cone voltages (V)	Collision voltages （V）
UGT1A1	β-estradiol-3-glucuronide	ESI (+)	471.3	199.0	50	20
UGT1A4	Trifluoperazine N-Glucuronide	ESI (+)	584.2	408.2	2	22
UGT2B7	3′-azido-3′-deoxythymidine-β-D-glucuronide	ESI (+)	466.2	290.1	14	16
IS_2 (+)	Phenacetin	ESI (+)	180.1	109.9	30	18
UGT1A3	Chenodeoxycholic acid 24-acyl-β-D-glucuronide	ESI (–)	567.3	112.9	50	26
UGT1A6	Serotonin-β-D-glucuronide	ESI (–)	351.1	112.9	22	16
UGT1A9	Propofol-β-D-glucuronide	ESI (–)	353.2	177.1	34	24
IS_2 (–)	Chlorpropamide	ESI (–)	275.0	189.9	30	18

Table S6. Mobile phase composition for determining metabolites of UGTs probe substrates.

Time(min)	Water-0.1% FA	Acetonitrile-0.1% FA
0.00	90	10
0.10	90	10
2.00	5	95
2.50	5	95
2.60	90	10
4.50	90	10

Supplementary Figures

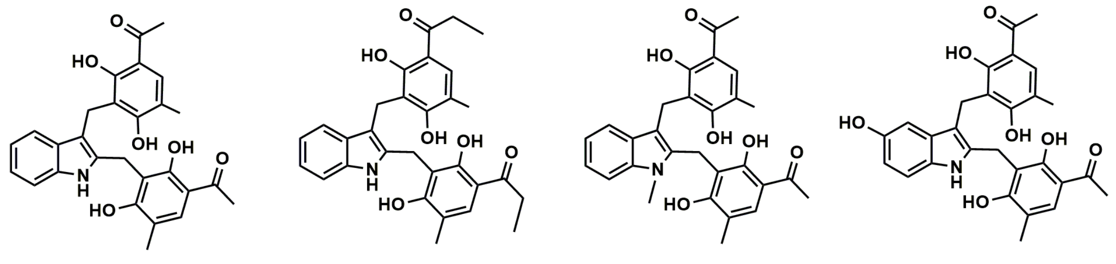

Figure S1. Chemical structures of penindolone and its derivatives HDYL-GQQ-2399, QL-Vir-09 and lbh3-78-2.

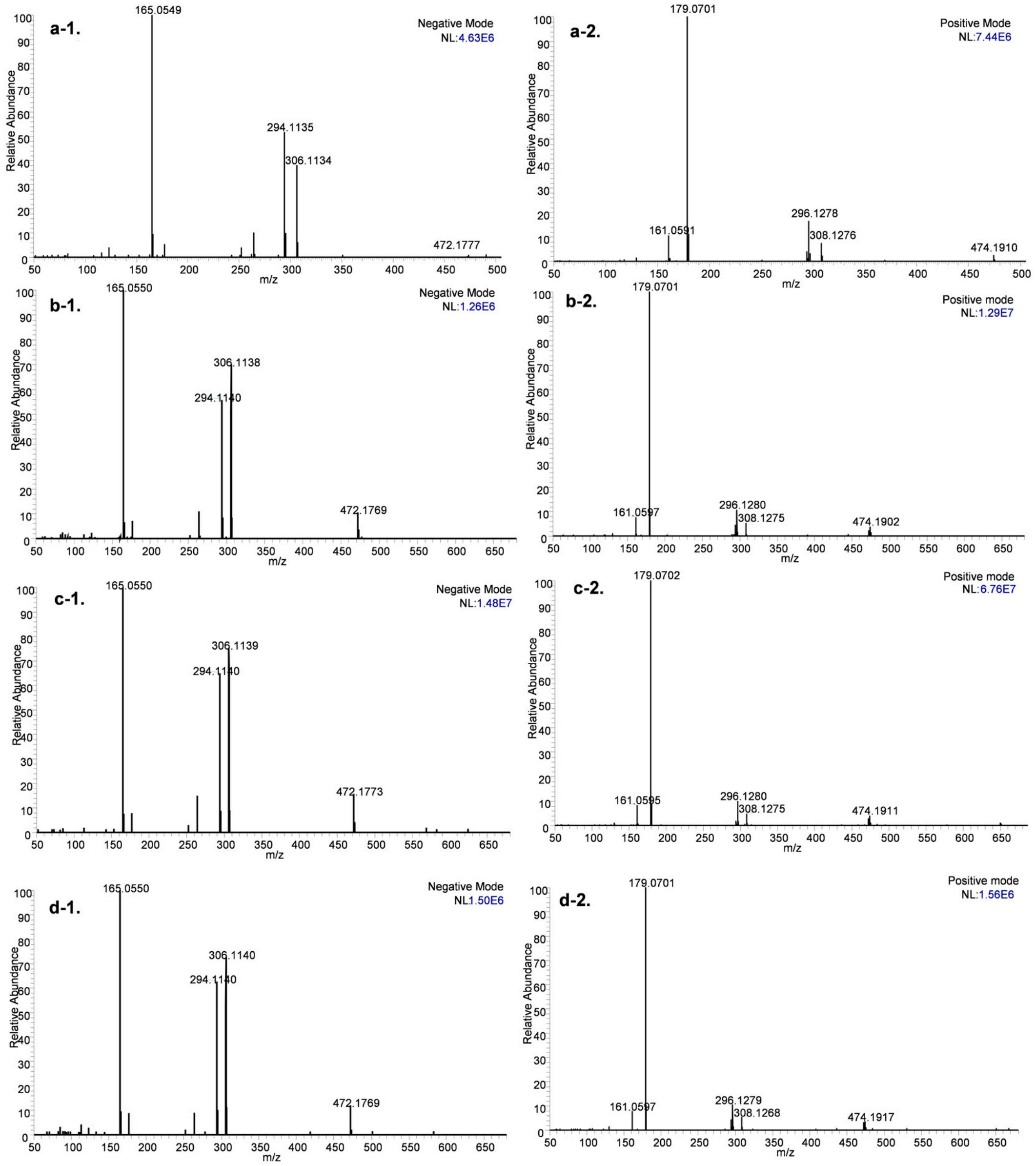

Figure S2. MS² spectra of M0 (**a-1**), M3 (**b-1**), M4 (**c-1**), M5 (**d-1**) obtained in negative mode and M0 (**a-2**), M3 (**b-2**), M4 (**c-2**), M5 (**d-2**) obtained in positive mode.

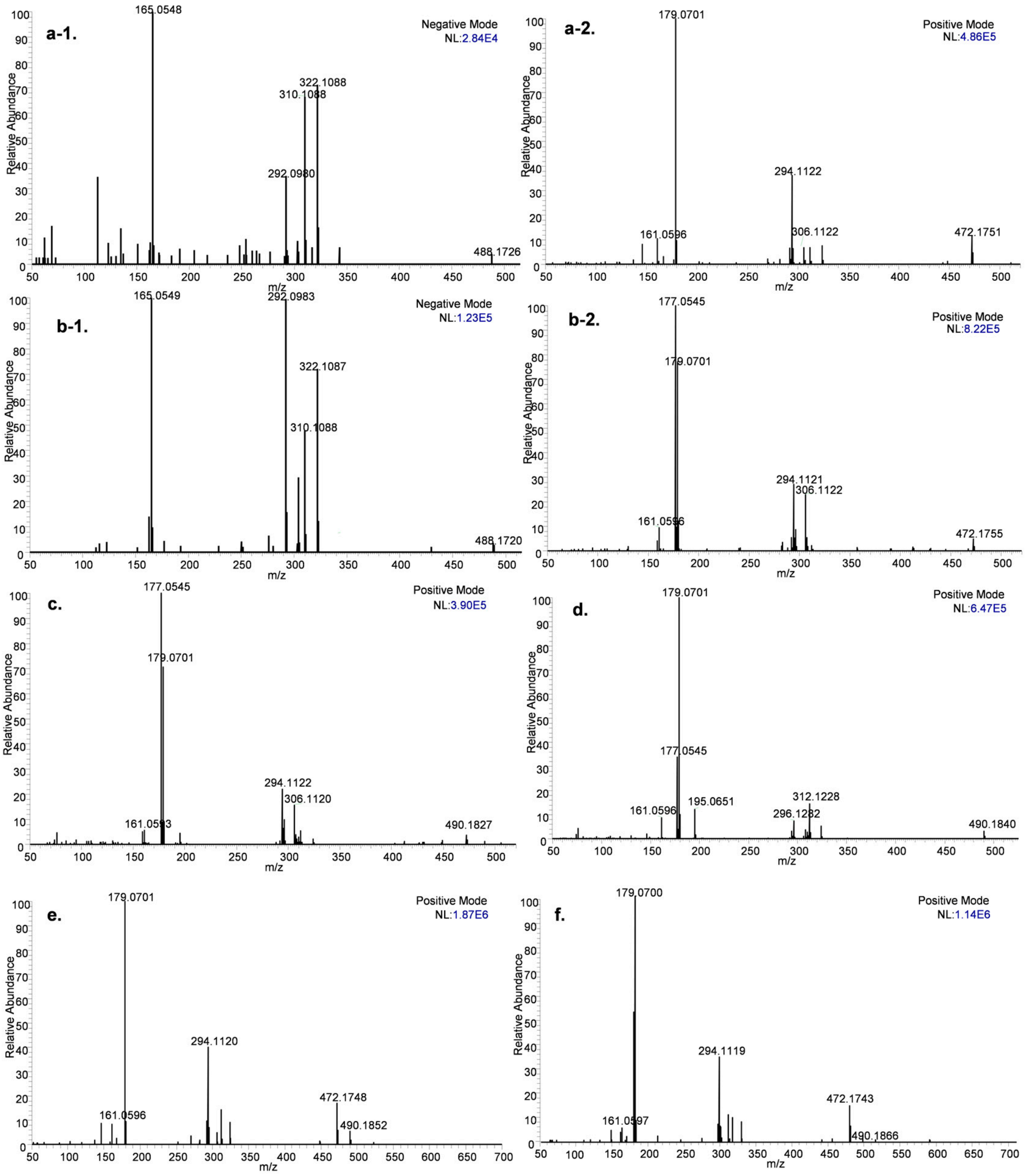

Figure S3. MS^2 spectra of M1 (**a-1** obtained in negative mode, **a-2** obtained in positive mode), M2 (**b-1** obtained in negative mode, **b-2** obtained in positive mode), M6 (**c**), M7 (**d**), M14 (**e**) and M15 (**f**).

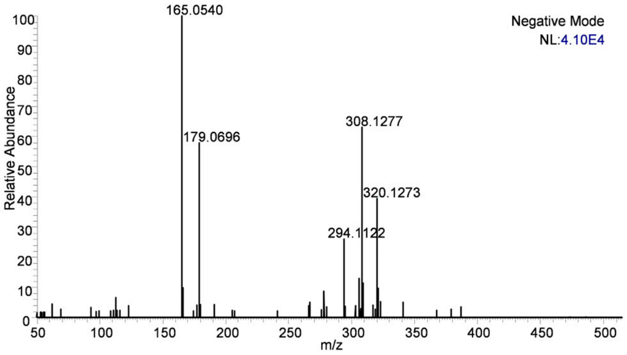

Figure S4. MS^2 spectra of M8.

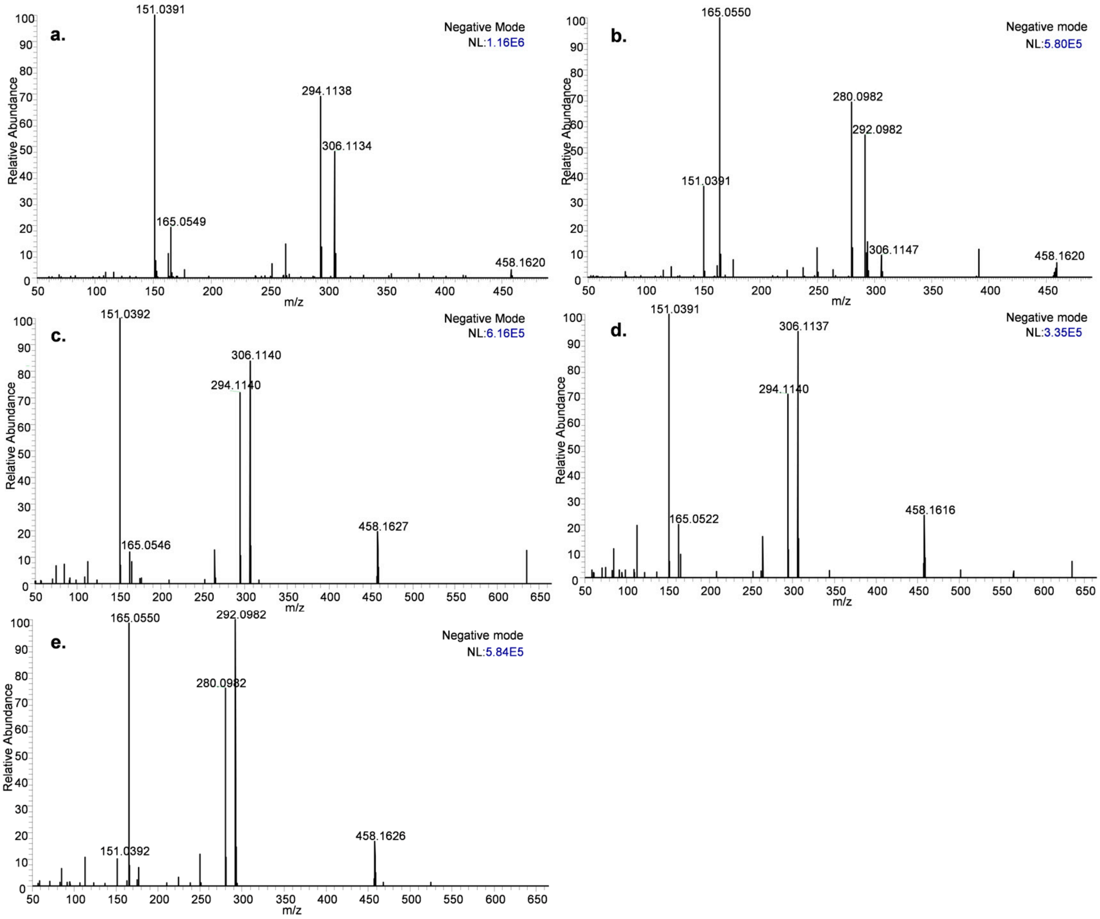

Figure S5. MS^2 spectra of M9 (**a**), M10 (**b**), M16 (**c**), M17 (**d**) and M18 (**e**).

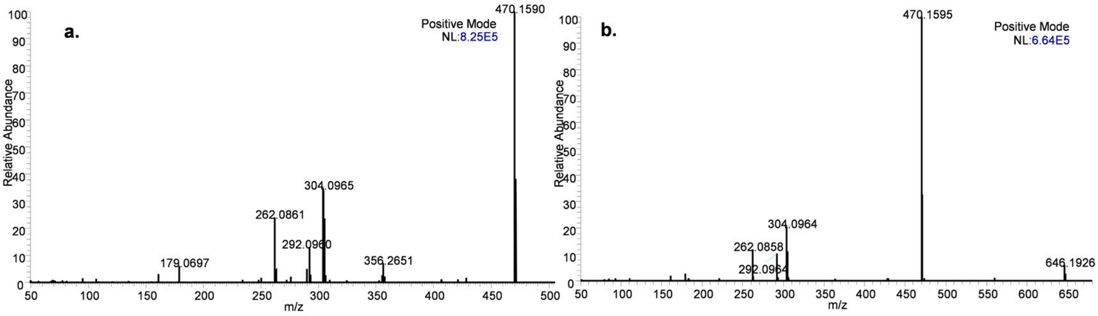

Figure S6. MS^2 spectra of M11 (**a**) and M19 (**b**).

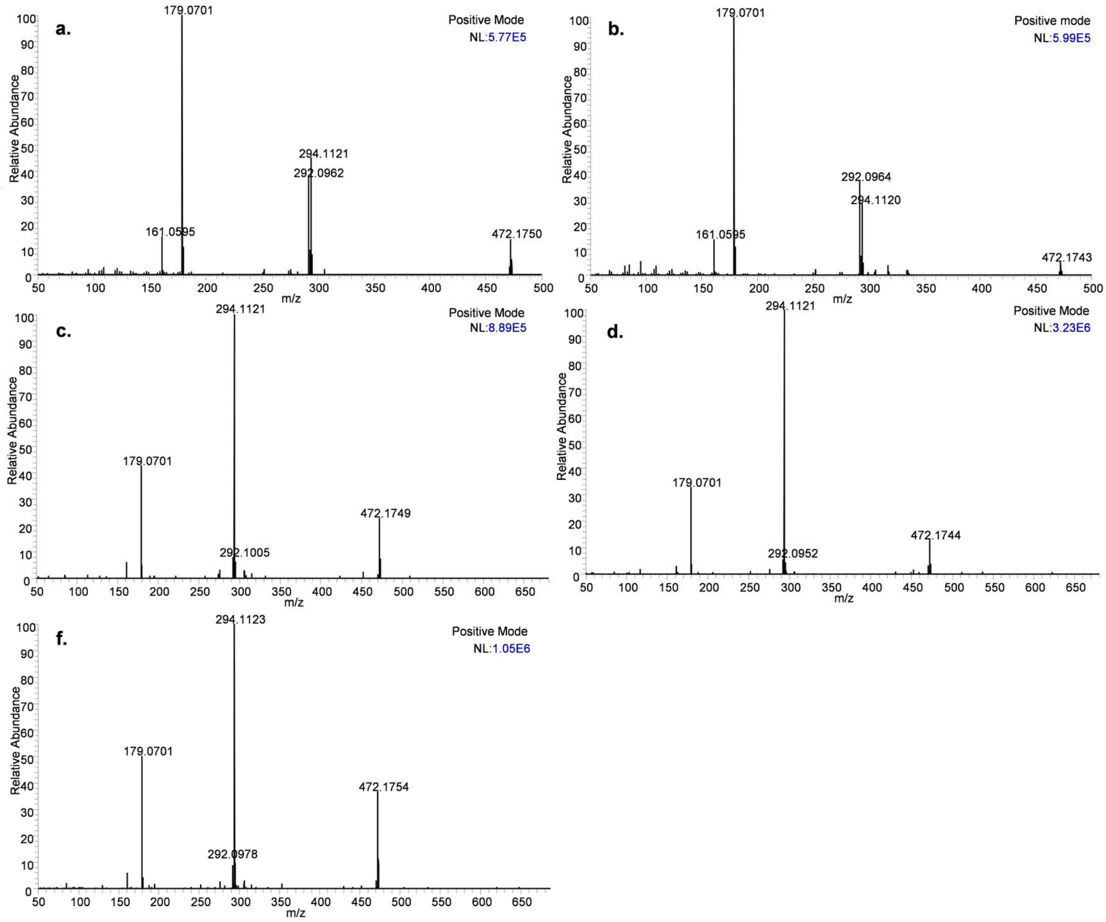

Figure S7. MS² spectra of M12 (**a**), M13 (**b**), M20 (**d**), M21 (**e**) and M22 (**f**).

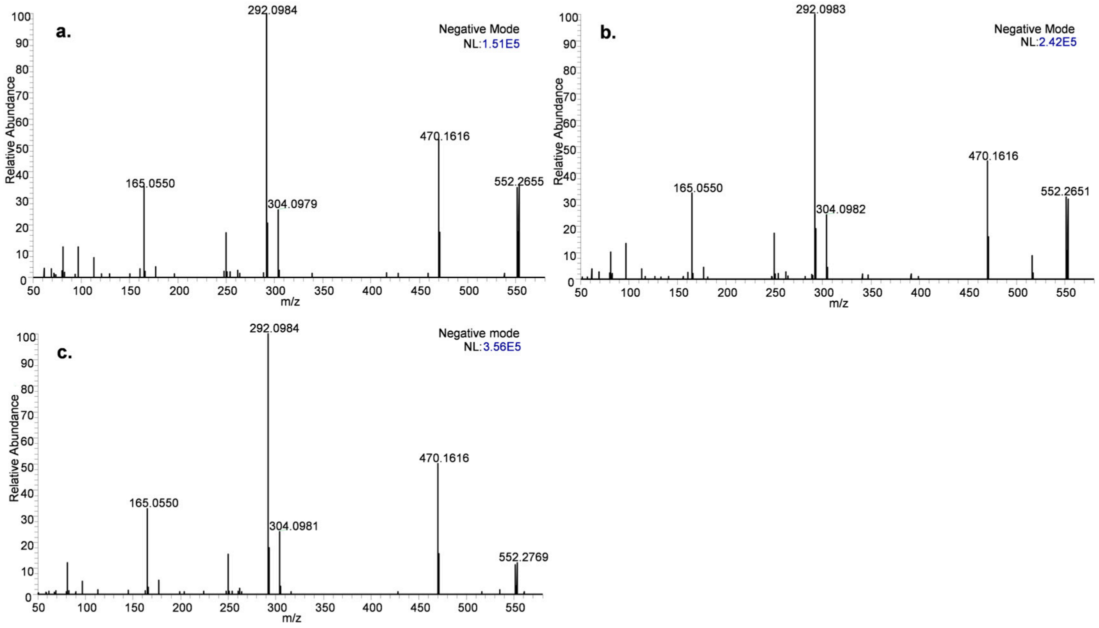

Figure S8. MS² spectra of M23 (**a**), M24 (**b**) and M25 (**c**).

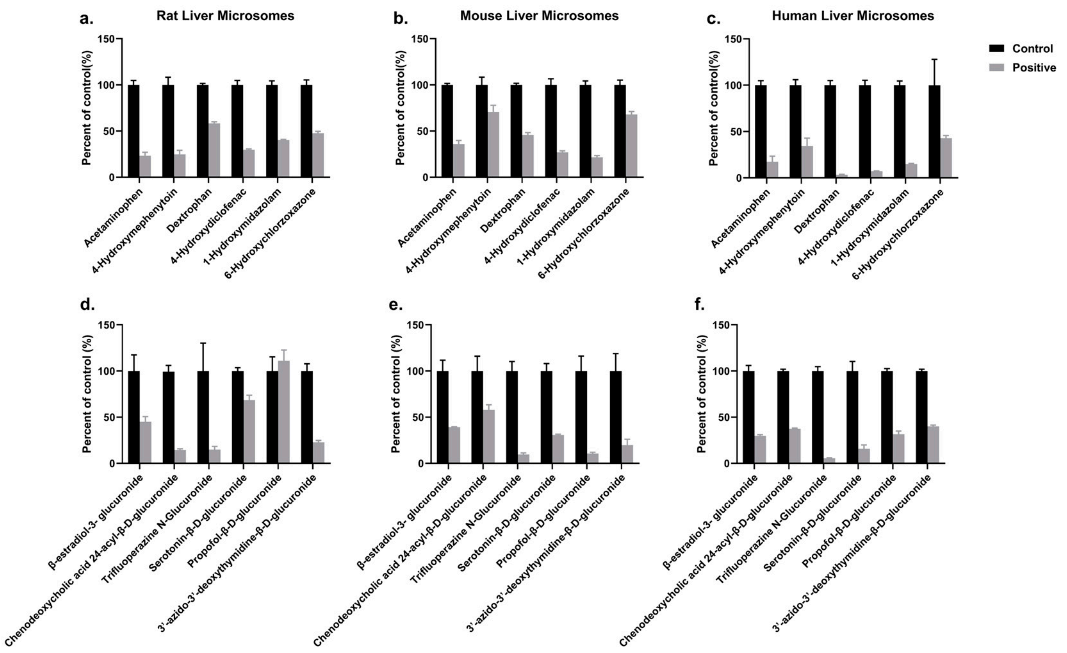

Figure S9. The validation of rat (**a**, **d**), mouse (**b**, **e**) and human (**c**, **f**) LMs system was confirmed by specific probe substrates. Error bars represent standard deviation.

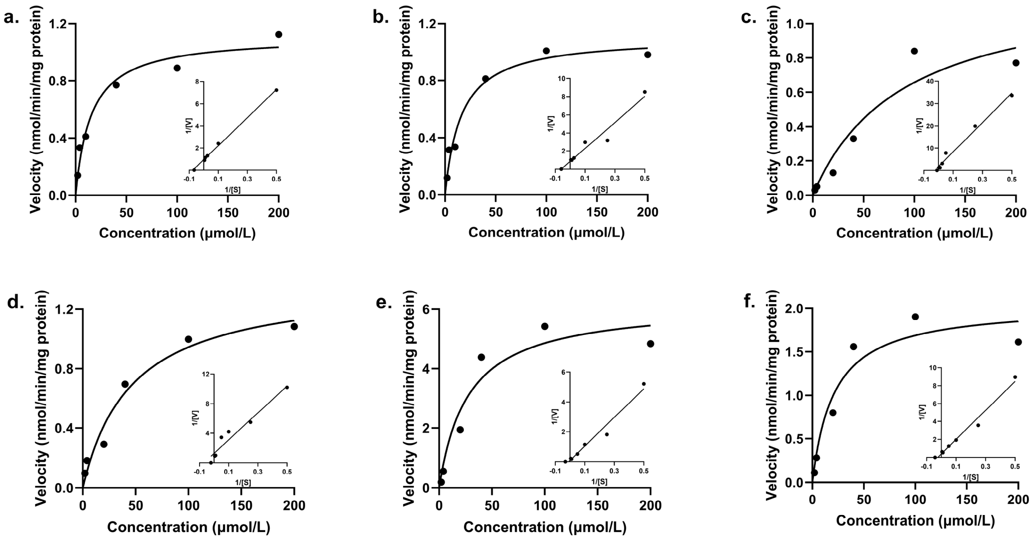

Figure S10. Enzyme (CYPs and UGTs) kinetic plots of PND metabolism in rat (**a**, **d**), mouse (**b**, **e**) and human (**c**, **f**) LMs. Data are shown as Michaelis-Menten plots. The insert shows the Lineweaver-Burk plot of 1/[S (μM)] versus 1/[V (nmol/min/mg protein)].
